# Effect of Tapentadol on Dogs’ Minimum Alveolar Concentration (MAC) of Isoflurane

**DOI:** 10.3390/ani15101381

**Published:** 2025-05-10

**Authors:** José Antonio Ibancovichi-Camarillo, Julio Raúl Chávez-Monteagudo, Marco Antonio De Paz-Campos, Lilia Gutiérrez-Olvera, Héctor Salvador Sumano-López

**Affiliations:** 1 Anestesiología y Analgesia, Facultad de Medicina Veterinaria, Universidad Autónoma del Estado de México, UAEMex, Toluca 50000, Mexico; ibanvet@gmail.com; 2 Departamento de Ciencias Pecuarias, Facultad de Estudios Superiores Cuautitlán, Hospital de Pequeñas Especies, Universidad Nacional Autónoma de México, UNAM, Cuautitlán Izcalli 54740, Mexico; 3 Departamento de Ciencias Biológicas, Facultad de Estudios Superiores Cuautitlán, Hospital de Pequeñas Especies, Universidad Nacional Autónoma de México, UNAM, Cuautitlán Izcalli 54740, Mexico; depaz_0@comunidad.unam.mx; 4 Departamento de Fisiología y Farmacología, Facultad de Medicina Veterinaria y Zootecnia, Universidad Nacional Autónoma de México, UNAM, Ciudad de México 04510, Mexico; liliago@unam.mx (L.G.-O.); sumano@unam.mx (H.S.S.-L.)

**Keywords:** tapentadol, isoflurane, dogs, μ-opioid receptor, norepinephrine

## Abstract

To evaluate the effect of oral administration of 10 mg kg^−1^ tapentadol on dogs’ minimum alveolar concentration of isoflurane. The MAC of isoflurane was determined in seven dogs without previous medication (Group MAC_ISO_) and 2 h 30 min after the administration of 10 mg kg^−1^ of tapentadol orally (Group MAC_ISO+TAP_). The MAC was determined by pinching the fourth finger of the pelvic limb. Data normality was analyzed using the Shapiro–Wilk test. Student’s t-test was used to evaluate the effect of tapentadol on isoflurane MAC and cardiovascular variables. The minimum alveolar concentration obtained in the Group MAC_ISO_ was 1.52 ± 0.02%, and in the Group MAC_ISO+TAP_, it was 1.47 ± 0.03%, observing a significant statistical difference between groups (*p* = 0.01). The reduction in the MAC caused by administering 10 mg kg^−1^ of Tapentadol was 3.29%. No statistically significant differences were observed when the heart rate, systolic blood pressure, diastolic blood pressure and mean arterial pressure were compared between groups. The oral administration of 10 mg kg^−1^ tapentadol reduces dogs’ minimum alveolar concentration of isoflurane without alterations in the heart rate and systemic blood pressure.

## Simple Summary

In this study, the effect of tapentadol on the minimum alveolar concentration (MAC) of isoflurane in dogs was evaluated. We observed that at the dose studied, even though there is statistical significance, the MAC only decreased by 3.29%. No changes were observed in the cardiovascular variables recorded.

## 1. Introduction

Tapentadol is an atypical opioid with a dual mechanism of action, it is a μ-opioid receptor agonist. It inhibits the reuptake of norepinephrine in the central nervous system [1], unlike other opioids, such as tramadol, meperidine, fentanyl and pentazocine, tapentadol is not associated with serotonin syndrome because it lacks serotonergic activity [2]. The analgesic effect of tapentadol does not depend on hepatic activation [3], as with tramadol, in which the analgesic effect is dependent on the formation of active metabolites by cytochrome P450 monooxygenase [4]. With a low bioavailability after the oral administration of only 4.4% [5], there are different reports on the analgesic effect of tapentadol in dogs [6,7]. On the other hand, opioids are considered the drugs of first choice in patients who have moderate to severe pain [8]; they are used for the control of acute pain in dogs and cats, such as in the case of trans-surgical and post-surgical pain, in trauma patients [9], and in sedation protocols and multimodal analgesia techniques since they are administered in conjunction with NSAIDs, alpha 2 adrenergic agonists, such as dexmedetomidine, and in combination with loco-regional analgesia techniques [10]; in this way, the requirements of anesthetic agents are reduced, allowing the necessary doses to be reduced to achieve the desired effect without the appearance of unwanted side effects, which decreases morbidity and mortality in patients under general anesthesia [11]. The minimum alveolar concentration (MAC) is the required alveolar concentration of an inhalation anesthetic necessary to prevent movement in 50% of subjects exposed to a supramaximal noxious stimulus [12,13]. The measurement of the MAC and its decrease because of analgesic drugs has been used as an objective measure of antinociception in individuals anesthetized with inhalation techniques [14]. The authors hypothesize that tapentadol administered at a dose of 10 mg kg^−1^ orally decreases the minimum alveolar concentration of isoflurane in dogs. If the hypothesis is confirmed and the reduction is clinically relevant, it will be important to adjust the doses of the inhalational anesthetic.

## 2. Materials and Methods

### 2.1. Animals

This study was submitted for review and approval by the Internal Committee for the Care and Use of Animals in Experimentation of the Faculty of Higher Studies of Cuautitlán of the UNAM (CICUAE-FESC) (experimental protocol number pC 22_11).

Seven adult mixed-breed dogs aged from 1 to 4 years, weighing 16.4 ± 4.7 Kg (four females and three males), were included. The medical history was reviewed to determine if the dogs were healthy, and a general physical examination, blood count, blood chemistry and urinalysis were performed. On the day of the experiment, food was only restricted 8 h before.

### 2.2. Experimental Design

In this prospective, blinded, randomized, crossover, experimental study, isoflurane MAC was measured in 7 dogs. The sample size was determined by statistical analysis of G* Power, which coincides with the sample size that has been used in different studies where the MAC has been calculated by other researchers [15,16]. Depending on the assigned experimental group, each dog was anesthetized twice and given treatment before MAC measurement. The experimental group assignment was performed using a Microsoft Excel 2024 random number generator. 

The experimental groups were as follows:

Group MAC_ISO_: The MAC of isoflurane was determined without prior medication (control group).

Group MAC_ISO+TAP_: A total of 10 mg kg^−1^ of tapentadol was administered orally 2 h 30 min before the measurement of the isoflurane MAC (experimental group). The dose administered in this investigation was determined from a study where the pharmacokinetics and pharmacodynamics of tapentadol were previously evaluated in three different doses [17].

Fifteen days were allowed to pass before re-anesthetizing and measuring the MAC in each dog.

### 2.3. Anesthetic Procedure

A 20-gauge catheter was placed in the cephalic vein (SAFELET, NIPRO MEDICAL LTDA, Sorocaba, Brazil) for the administration of the propofol (inducing drug) as a single dose and for the administration of 4 mL kg hr^−1^ of 0.9% saline solution (Pisa Agropecuaria, Guadalajara Jalisco, Mexico) throughout the anesthetic procedure using an infusion pump (BeneFusion VP1, Gu Vet, Shenzhen, China). Induction was performed by administering propofol to effect intravenously at a dose of 4–6 mg kg^−1^ (Xenprof; Aculife Healthcare PVT, Ltd., Village-Sachana, Tal-Viragam, India). All dogs were endotracheally intubated using an appropriately sized tube and connected to a rebreathing system at an anesthetic station (Mindray Animal Care, Veterinary Anesthesia Machine, WATO EX35Vet, Shenzhen, China). Anesthetic maintenance was performed by administering isoflurane (ISO) (Sofloran Vet, Isoflurane, PiSA Agropecuaria, S.A. de C.V. Guadalajara, Jalisco, Mexico) vaporized in 100% oxygen with a fresh gas flow of 100 mL kg^−1^ min^−1^ for the first 10 min and then decreased to a flow of 50 mL kg^−1^ min^−1^. The lungs were mechanically ventilated using an anesthetic station using volume-controlled ventilation, adjusting the respiratory rate and airway pressure to maintain eucapnia (exhaled carbon dioxide) Et_CO2_ (35–45 mmHg). Dogs were positioned in lateral recumbency, and a catheter was placed in the dorsal pedal artery for continuous monitoring of systolic blood pressure (SAP), diastolic blood pressure (DAP), and mean arterial pressure (MAP) via a blood pressure transducer (Transducer Macrodrip, Icumedical, San Clemente, CA, USA). Before each experiment, the transducers were zeroed to atmospheric pressure, calibrated using a mercury manometer, and leveled at heart level. An esophageal thermometer was placed to monitor body temperature (T°), which was maintained between 37.5 and 39.2 °C using a warm air circulation system (Veterinary Automatic Air Warming System, Hefei Longshore Tech Co., Ltd., Hefei City, China). Electrocardiography through lead II constantly measures the heart rate (HR) and rhythm. A pulse oximetry sensor was placed on the tongue to measure saturation (SpO_2_). An infrared gas analyzer was used to continuously measure inspired (Fi_ISO_) and expired isoflurane (Fe_ISO_) concentrations, Et_CO2_ and the respiratory rate (RR). Et_CO2_, SAP, DAP, MAP, T°, HR, SpO_2_, Fi_ISO_ and Fe_ISO_ were monitored with a Mindray ePM 12 M Vet monitor (Shenzhen Mindray Animal Medical Technology Co., Ltd, Guanlan Street, Longhua District, Shenzhen, China). The anesthesia monitor was calibrated each morning using a calibration gas specifically designed for this purpose (DOT-34 NRC 300/375M1014; Datex-Ohmeda Division, Helsinki, Finland).

### 2.4. MAC Determination

The determination of the MAC of isoflurane was performed after a 60 min equilibration period to eliminate the effect of propofol [15] at an Fe_ISO_ of 1.6% [16] The MAC of isoflurane was measured in patients without previous treatment, which was named Group MAC_ISO_; similarly, in the Group MAC_ISO+TAP,_ the MAC was determined in dogs to which tapentadol was administered orally at a dose of 10 mg kg^−1^, 2 h 30 min before anesthetic induction [17].

The technique used to determine the MAC was standardized and previously reported by other researchers and consists of applying a noxious stimulus by compressing the fourth finger of the pelvic limbs with 24 cm long forceps to which a sponge was placed in each jaw to avoid tissue damage, closing the piece only up to the first notch [18]. The stimulus must be maintained for 60 s and stopped if a positive response is observed [19]. A positive response is considered if the dog moves its head, chest, abdomen or limbs. A negative response was determined if no head, thorax, abdomen or tail movement was observed and if only muscle rigidity, swallowing or ventilatory efforts were present. If a positive response was observed, Fi_ISO_ was increased by 10% and maintained at that concentration for 15 min to allow equilibration between fractions. In case of a negative response, Fi_ISO_ was reduced by 10%, and this concentration was maintained for 15 min before the stimulus was applied again. The procedure was repeated until a positive response was obtained. The MAC was calculated on two occasions in each dog [19]. Each dog’s MAC was considered the mean value between the lowest concentration that allows movement and the highest concentration that inhibits it after applying the stimulus. The person (the anesthesiologist) who evaluated the response to the painful stimulus always did it without knowing to which experimental group each individual belonged. The HR, SAP, DAP and MAP were recorded before each stimulus. Because the experiment was performed in Cuautitlán Izcalli, State of Mexico, where the barometric pressure is 580 mmHg, isoflurane MAC values were corrected to sea level pressure by dividing 580/760 mmHg and multiplying by the MAC value obtained. After determining the MAC, the vaporizer was closed, and the dogs were kept connected to the anesthetic circuit, always monitoring the beginning of the ventilatory efforts to suspend controlled ventilation to allow spontaneous ventilation and disconnect the dog from the anesthetic circuit, extubating it when a swallowing reflex was observed. After each experiment, all dogs were administered 0.2 mg kg^−1^ of meloxicam (Melocaxyl. Meloxicam, PiSA Agropecuaria, S.A. de C.V. Guadalajara, Jalisco, Mexico) subcutaneously, and 24 h later, they were administered meloxicam for the second time at a dose of 0.1 mg kg^−1^ orally.

### 2.5. Statistical Analysis

Statistical analysis was performed using GraphPad Prism, Version 10.4.2 (534) Inc., USA. Data normality was analyzed by applying the Shapiro–Wilk test. A paired *t*-test was used to evaluate the effect of tapentadol on isoflurane MAC and cardiovascular variables. We reported the values as mean ± SD. Statistical significance was considered if the values of *p* < 0.05.

## 3. Results

The dose of propofol administered in both groups was 5.7 ± 0.48 mg Kg^−1^; there is no statistical difference when comparing them. The minimum alveolar concentration obtained in Group MAC_ISO_ was 1.52 ± 0.02%, and in Group MAC_ISO+TAP_, it was 1.47 ± 0.03%, observing a significant statistical difference between groups (*p* = 0.01). The reduction in the MAC caused by the administration of tapentadol was 3.29%. Table 1 shows the values of the isoflurane MAC, HR, SAP, DAP, MAP, the dose of propofol administered, the time that elapsed to determine the MAC, the time to extubation after closing the isoflurane vaporizer of Group MAC_ISO_ and the MAC of Group MAC_ISO+TAP._ When comparing the heart rate, systolic arterial pressure, diastolic arterial pressure and mean arterial pressure of Group MAC_ISO_ with the Group MAC_ISO+TAP_, no statistically significant differences were observed. The time for MAC determination in the Group MAC_ISO_ was 159.4 ± 7.06 min, and in the Group MAC_ISO+TAP_, it was 182.9 ± 6.46 min, observing a significant statistical difference between groups (*p* = 0.0001). The time to extubation after closing the isoflurane vaporizer of Group MAC_ISO_ was 10.59 ± 1.35, and in the Group MAC_ISO+TAP_, it was 10.26 ± 1.37, with no statistically significant differences observed.

## 4. Discussion

In this study, we can observe that administering 10 mg kg^−1^ of tapentadol orally 2 h and 30 min before anesthesia with isoflurane decreases the minimum alveolar concentration of isoflurane in dogs by 3.29%. Despite observing a slight decrease in the MAC, the applied statistical analysis shows statistical significance, confirming our initially stated hypothesis. Caution should be exercised with these results, since the reduction is small, and its clinical application does not seem to be relevant, and the effect of variation that could occur between individuals could be a factor to consider in this small reduction. Tapentadol at a dose of 10 mg kg^−1^ marginally reduces the MAC of isoflurane, without being clinically important, and could be part of the variation between individuals.

Similarly, the MAC measurement of isoflurane was performed 2 h and 30 min after oral administration, based on the work where the pharmacokinetics of tapentadol are reported [17] and considering the 60 min wait during which the effect of the propofol is eliminated [15]. The mechanism involved in the decrease in the MAC by tapentadol can be attributed to its action as an agonist of the opioid receptor µ and the inhibition of norepinephrine reuptake [1].

On the other hand, the effect of opioid analgesics on the MAC of halogenated anesthetics has been used as a measure of their analgesic efficacy [14]; however, there are also reports of the effect of non-analgesic drugs on the MAC from inhalation anesthetics [20]. There are reports of the analgesic effect of the administration of tapentadol for pain control in an experimental model in which a nociceptive effect more remarkable than that of tramadol and like that of morphine is mentioned [6]; however, it should be considered that the administered dose of tapentadol was lower than the one we administered in our studio, but the administration route was intravenous. Similarly, researchers have already studied the use of tapentadol for pain control due to cranial cruciate ligament rupture, where an improvement was observed in the subjective evaluation of claudication but not in the objective assessment [7]. In this study, the administered dose was 30 mg kg^−1^ orally, three times higher than in our research. It will be essential to evaluate the 30 mg kg^−1^ dose on dogs’ minimum alveolar concentration of isoflurane. Considering tapentadol has little effect at a dose of 10 mg kg^−1^ on the MAC of isoflurane in dogs, the dose tested here is too low to be indicated for a painful process. This coincides with the higher dose and the sound effect observed as an analgesic in cranial cruciate ligament rupture, considering the decrease in MAC as an objective measure of analgesic effect. Likewise, tapentadol has been used as an analgesic for the control of chronic pain in horses; therefore, the present study represents the beginning of the evaluation of the effect generated by tapentadol and its interaction with other drugs in case this opioid begins to be used as an analgesic for the control of chronic pain in dogs [21]. We also need to highlight that the dogs involved in the study were healthy and did not have any painful conditions, limiting the transfer of the results to a clinical context where tapentadol could be prescribed as an analgesic. Pain models will need to be considered to assess the effect of tapentadol on isoflurane MAC.

No differences were observed in the required doses of propofol between groups. MAC measurement times were longer in the MAC_ISO+TAP_, and there was no difference between the times to extubation.

The MAC of isoflurane without the effect of any treatment coincides with what other authors previously reported [20,21,22].

When evaluating the cardiovascular parameters of dogs anesthetized with isoflurane alone, we observed no effect on the heart rate in conjunction with tapentadol, coinciding with what was previously reported [17]. To the authors’ knowledge, the present study is the first to report the impact of tapentadol on systemic blood pressure. No significant differences were observed in dogs who were orally given 10 mg kg^−1^ of tapentadol compared to dogs given no medication.

This study is not designed to determine whether the reduction in the MAC by tapentadol is dose-dependent. It should be mentioned that the dose of tapentadol used in this experiment was considered from a study in which plasma concentrations of tapentadol were measured from three different doses (10, 20 and 30 mg kg^−1^), where it was determined that the plasma concentrations were dose-dependent [17]; therefore, the same could happen with the dose of tapentadol and its effect on the MAC of isoflurane in dogs; it could be dose dependent. Studies will be necessary in this regard. It is important to mention that in this research, a single dose of tapentadol was evaluated (10 mg kg^−1^), and a dose–response test was not performed; therefore, it is necessary to carry out future studies to evaluate relevant clinical effects with different doses.

It is also necessary to comment that plasma measurements of tapentadol and its mostly active metabolite [17] were not performed in the present study, which could be desired when determining the MAC.

## 5. Conclusions

The oral administration of 10 mg kg^−1^ tapentadol reduces dogs’ minimum alveolar concentration of isoflurane, without alterations in the heart rate and systemic blood pressure. This study could be a pilot study on the use of tapentadol in dogs that will undergo inhalation anesthesia. However, this reduction is very small; clinically, it will not be significant; therefore, more research is required with different doses of tapentadol to know if the reduction in isoflurane MAC is dose-dependent and thus be able to determine its clinical application.

## Figures and Tables

**Table 1:** The minimum alveolar concentration of isoflurane (mean ± standard deviation) of 7 dogs anesthetized with isoflurane alone or in combination with tapentadol at a dose of 10 mg Kg^−1^ orally administered 2 h 30 min before the anesthetic procedure.

Variable	Isoflurane	Isoflurane + Tapentadol
Isoflurane MAC (%)	1.52 ± 0.02	1.47 ± 0.03 *p <* 0.011 *
HR (beats per minute)	117.3 ± 8.95	112 ± 10.89 *p <* 0.296
SAP (mm Hg)	111 ± 7.34	115.8 ± 8.6 *p <* 0.310
DAP (mm Hg)	62.5 ± 3.56	66.83 ± 3.76 *p <* 0.332
MAP (mm Hg)	78.67 ± 3.72	83 ± 5.02 *p <* 0.092
Propofol dose mg Kg^−1^	5.7 ± 0.48	5.7 ± 0.48 *p <* 0.99
MAC determination (min)	159.4 ± 7.06	182.9 ± 6.46 *p <* 0.0001 *+*
Time to extubation (min)	10.59 ± 1.35	10.26 ± 1.37 *p <* 0.660

MAC (minimum alveolar concentration), HR (heart rate), SAP (systolic arterial pressure), DAP (diastolic arterial pressure) and MAP (mean arterial pressure). Statistical significance was considered if the values of *p <* 0.05. * *p* value = 0.011. + *p* value = 0.0001.

## Data Availability

The data presented in this study are available on request from the corresponding author.
